# African Resources and the Promise of Resilience against COVID-19

**DOI:** 10.4269/ajtmh.20-0470

**Published:** 2020-06-03

**Authors:** Ronald E. Blanton, Nancy B. Mock, Honelgn N. Hiruy, John S. Schieffelin, Seydou Doumbia, Christian Happi, Robert J. Samuels, Richard A. Oberhelman

**Affiliations:** 1 Tulane School of Public Health and Tropical Medicine, New Orleans, Louisiana;; 2 Tulane School of Medicine, New Orleans, Louisiana;; 3 University Clinical Research Center (UCRC), University of Sciences, Techniques and Technology of Bamako (USTTB), Bamako, Mali;; 4 African Center of Excellence for Genomics of Infectious Diseases (ACEGID) Redeemer’s University, Ede, Nigeria;; 5 National Lassa Fever Program, Ministry of Health and Sanitation, Kenema Government Hospital, Kenema, Sierra Leone

The COVID-19 pandemic has been slow to arrive in the world’s poorest countries, especially in Africa. There are good reasons to believe that the consequences for the continent could be worse than anywhere else.^1–3^ The weaknesses of some governments, healthcare systems, and economies, plus armed conflict, are factors that the virus can and will exploit. A recent British Broadcasting Corporation article noted that there are 10 African countries that have no ventilators, nine have < 1 per 1 million people, and most of the others have too few to serve their populations in an outbreak of U.S. proportions.^2^ African countries need help but are not all helpless. To adequately preview the impact of COVID-19 on the continent, however, both weaknesses and strengths must be considered. The Africa of 2020 is not the Africa of 1960 or even 2014. Africa is a continent of 54 countries, with a range of climatic, cultural, demographic, and economic conditions that contrast them with more developed regions and with each other (Table 1). The country-to-country effects of COVID-19 could be quite different, and there are resources that may help produce better than expected outcomes.

**Table 1:** Characteristics that may influence COVID-19 outcomes of selected sub-Saharan countries

	Pop. (×10^6^)*	Age > 65 years (%)*	Diabetes (%)†	BMI > 25 (%)‡	Urban (%)*	GDP/capita (US$)*	Health spending/capita (US$)*	Service coverage§	Beds/100 K‡	MDs/100 K‖	Nurses/100 K‖
Angola	31	2	5	24	55	3,432	114	40	80	22	41
Burkina Faso	20	2	7	20	40	715	44	40	40	9	88
Cotê d’Ivoire	84	3	2	29	66	1,716	70	47	40	23	60
DR Congo	25	3	6	22	29	562	19	41	80	7	111
Eq Guinea	1	2	6	25	44	10,262	301	45	210	40	50
Ethiopia	109	4	4	18	51	772	25	39	30	8	71
Ghana	30	3	3	29	72	2,202	67	47	90	14	235
Kenya	51	2	3	23	21	1,711	77	55	140	16	117
Liberia	5	3	2	28	56	677	57	39	80	4	53
Niger	22	3	2	20	27	414	29	37	30	4	27
Nigeria	196	3	3	26	51	2,028	74	42	50	38	118
Rwanda	12	3	5	22	16	773	49	57	160	13	120
South Africa	58	5	13	52	50	6,374	499	69	280	91	131
Sub-Saharan Africa	1,078	3	5	–	17	1,586	78	–	91	–	–
United States	327	16	11	68	82	62,795	10,246	84	290	261	1,455
Italy	60	23	5	64	70	34,483	2,840	82	340	398	574
China	1,393	11	9	34	59	9,771	441	79	420	198	266
World	7,594	9	8	–	66	11,313	913	–	270	–	–

BMI, body mass index; GDP, gross domestic product.

* 2018 Worldbank. World Development Indicators. https://databank.worldbank.org/indicator/NY.GDP.PCAP.CD/1ff4a498/Popular-Indicators#.

† 2019 International Diabetes Foundation. Age-adjusted rates. https://www.diabetesatlas.org/en/.

‡ 2015–2019 WHO. The Global Health Observatory. https://www.who.int/data/gho/data/indicators.

§ 2017 WHO. The Global Health Observatory. https://apps.who.int/gho/portal/uhc-cabinet-wrapper-v2.jsp?id=1010501. Service coverage is an index composed of measures of financial risk protection, access to quality essential healthcare services, and access to safe, effective, quality, and affordable essential medicines and vaccines. Scored 0–100.

‖ 2014–2018 WHO World Health Statistics data visualizations dashboard. https://apps.who.int/gho/data/node.sdg.3-c-data?lang=en.

Demographics: The population structure in Africa is relatively protected against serious COVID-19. The major risk factors for a serious disease are older age, diabetes, heart disease, and obesity.^4,5^ Africa is young. Only 2–5% of Africans are older than 65 years, and the median age is 19 years. Rates of chronic diseases, although rising, are currently lower than those of any other continent (Table 1). Sub-Saharan Africa is still mostly rural (59%). This is rapidly changing, but rural areas are less densely populated, and mobility in many areas is limited by poor road and air transportation. Thus, some physical distancing is built into the structure of many countries. On the other hand, urban areas are densely packed, and poverty can be extreme. A major portion of the population will not eat if they must shelter at home. That home in Nairobi’s Kibera slum, for example, is in a community with 300,000 people per square kilometer (New York City has 10,000 per square kilometer). These are places where physical distancing has no meaning.

Healthcare systems: In comparison with the United States, Italy, and China, healthcare systems are fragile in most countries on the continent (Table 1). Scarcity of healthcare workers is one of the weakest points across all African countries. South Africa and Ghana, however, have more than 200 hospital beds per 100,000 populations, which compares favorably with the United States. Not reflected in the numbers are recent initiatives in healthcare management by governments. Several countries have instituted national health insurance programs in an effort to achieve near-universal coverage.^6^ Ethiopia aims to achieve universal coverage of primary care with new construction, equipment, and community-based health insurance for most of its population. Rwanda also provides near-universal health insurance through a community-based system. Finally, African countries have substantial experience in epidemic management and contact-tracing because of their experience with viral hemorrhagic fever epidemics, cyclical meningitis, outbreaks of cholera, and the high prevalence of HIV and tuberculosis.

Regional economic communities: To partially offset their individual economic vulnerabilities, African countries excel in cooperative efforts that provide additional economic resilience. The African Union seeks to promote cooperation for development built on the foundation of eight regional institutions, covering all countries on the continent. Public health is a priority area, and, in the context of the pandemic, each of the regional institutions works to distribute personal protection equipment (PPE) and diagnostic kits, pool procurement services, monitor socioeconomic impact, provide daily updates, help member states adopt WHO guidelines and mobilization strategies, and exchange information through National Emergency Operations Centers. The West African Health Organization and the African CDC also facilitate information-sharing, training, and provision of personnel. Other public–private efforts include the contribution of PPE and test kits by China’s Jack Ma Foundation and Ethiopia’s Prime Minister Abiy Ahmed.^7^ Nigeria’s Private Sector Coalition Against COVID-19 (CACOVID^8^) has contributed about US$100 million to support the Nigerian government’s pandemic response.

Biomedical capacity: Many countries in Africa have the intellectual resources and incipient infrastructure to conduct molecular surveillance, thanks to international cooperative programs led by the health and agricultural sectors. Benin, Burkina Faso, Cameroon, Ghana, Nigeria, Senegal, and Togo obtained large loans from the World Bank to establish the African Centers of Excellence Project. Some of these centers have been in the forefront of governmental response to outbreaks like Lassa fever, monkeypox, yellow fever, and, presently, COVID-19. The Redeemer University African Center of Excellence for Genomics of Infectious Disease and the West African Centre for Cell Biology of Infectious Pathogens in Ghana represent just two of these. Both sequenced African strains of the novel coronavirus soon after cases appeared. Elsewhere on the continent, examples are the Centre International de Recherche Médicale de Franceville in Gabon, the Sub-Saharan African Network for tuberculosis (TB)/HIV Research Excellence in South Africa, and the Mali University Clinical Research Center program (UCRC-SerefoLab). Starting in 2010, the WHO established BSL3 laboratories in at least 11 countries south of the Sahara for the diagnosis and study of dangerous pathogens through the Emerging and Dangerous Pathogens Laboratory Network. Many more outstanding African institutions are in place across the continent.

Containment and mitigation: There is evidence that as the epidemic evolves in Africa, it may be following a different playbook than that in other regions, in the context of numerous demographic, cultural, and political differences that exist in this least developed continent. Still, African countries are at risk for catastrophic health system failures if even modest pressure is put on their healthcare resources. Rosenthal et al.^9^ have pointed to many of the challenges to African countries facing the COVID-19 pandemic and steps that should be taken to mitigate its effect. The local resources pointed to here suggest that impacts will be less than what would have been expected even a decade ago. Given the resilience that exists in many of these countries, what needs to be done, and what is the role of the rest of the globe in this global emergency? African testing capacity is uneven across countries and should be supported with donations of kits and expertise. Testing and control efforts should be more decentralized and even made mobile to reach the largest number of people. Intensive care capacity needs support and expansion in facilities, equipment, and trained personnel to increase the overall quality of care. Lifesavers at all levels of care will be generators and renewable energy sources to supplement unreliable electricity. Lessons learned from specific experience with the COVID-19 epidemic should be gifted to Africa from more developed countries, where the experience has been longer and more intense to this point. In the long term, African societies will also need support to address the economic impacts of efforts to contain and mitigate the epidemic.
